# Functional Magnetic Resonance Imaging for Imaging Neural Activity in the Human Brain: The Annual Progress

**DOI:** 10.1155/2012/613465

**Published:** 2012-01-26

**Authors:** Shengyong Chen, Xiaoli Li

**Affiliations:** ^1^College of Computer Science and Technology, Zhejiang University of Technology, Hangzhou 310023, China; ^2^National Key Laboratory of Cognitve Neuroscience and Learning, Beijing Normal University, Beijing 100875, China

## Abstract

Functional magnetic resonance imaging (fMRI) is recently developed and applied to measure the hemodynamic response related to neural activity. The fMRI can not only noninvasively record brain signals without risks of ionising radiation inherent in other scanning methods, such as CT or PET scans, but also record signal from all regions of the brain, unlike EEG/MEG which are biased towards the cortical surface. This paper introduces the fundamental principles and summarizes the research progress of the last year for imaging neural activity in the human brain. Aims of functional analysis of neural activity from fMRI include biological findings, functional connectivity, vision and hearing research, emotional research, neurosurgical planning, pain management, and many others. Besides formulations and basic processing methods, models and strategies of processing technology are introduced, including general linear model, nonlinear model, generative model, spatial pattern analysis, statistical analysis, correlation analysis, and multimodal combination. This paper provides readers the most recent representative contributions in the area.

## 1. Introduction

Functional magnetic resonance imaging (functional MRI or fMRI) is based on the increase in blood flow to the local vasculature that accompanies neural activity in the brain. This results in a corresponding local reduction in deoxyhemoglobin because the increase in blood flow occurs without that similar magnitude in oxygen extraction. Deoxyhemoglobin is paramagnetic, and it alters the weighted MRI signal and thus is sometimes referred to as an endogenous contrast-enhancing agent. It also serves as the source of the signal for fMRI. Using an appropriate imaging sequence, human cortical functions can be identified without the use of exogenous contrast-enhancing agents on a clinical strength scanner. It has been confirmed that functional activity of the human brain from the MR signal is in anatomically distinct areas in the visual cortex, the motor cortex, and Broca's area of language-related activities. For example, Stroop test is commonly used as a behavior-testing tool for psychological examinations that are related to attention and cognitive control of the brain [1].

Over 100 years ago, it has been known that changes in blood flow and blood oxygenation (i.e., hemodynamics) are closely linked to neural activities in the brain. When neural cells are active, they increase the consumption of energy from glucose and switch to less energetically effective, but more rapid anaerobic glycolysis. The local response to this energy consumption is to increase blood flow to regions of increased neural activity, which occurs after a 1-2-second delay. The hemodynamic response rises to a peak over 4–6 seconds, before falling back to its baseline. This leads to changes in local cerebral blood volume and local changes in the concentration of oxyhemoglobin, which are detectable through the paramagnetic effects [2].

fMRI is highly interdisciplinary, and many studies are from several different fields, for example, physics (underlying fMRI signals and understanding of the principles), psychology (cognitive psychological, cognitive psychophysiological, and psychophysical experiments for obtaining extra measurements in addition to behavioral or electroencephalographic measurements), neuroanatomy (linking fMRI signals to understanding of the neuroanatomy), statistics (for correct observations and avoiding false-positive results), and electrophysiology (neuronal behavior at the electrophysiological level) [3].

In early 1990s, it has been recognized the potential importance of blood-oxygen-level dependence (BOLD), which is the MRI contrast of blood deoxyhemoglobin, for functional brain imaging with MRI. The first successful fMRI study was reported in *Science* journal by Belliveau et al. in 1991 [4]. Now fMRI has come to dominate the brain mapping field due to its relatively low invasiveness, absence of radiation exposure, and relatively wide availability [3]. Further, rapidly emerging studies correspond findings between fMRI and conventional electrophysiological techniques to locate specific functions of the brain [5]. Consequently, the number of medical and research centers with fMRI capabilities and investigational programs continues to escalate [2]. Now BOLD-based fMRI becomes a powerful tool for studying brain function not only locally but also on the large scale [6]. The particular imaging methods and procedures vary from every individual institute. Yet there is no completely standardized package of software for clinical use.

Although the current fMRI uses BOLD as the method for determining active areas as the result of various experiences, the signals are relative and not individually quantitative. The recent fMRI technology extends traditional anatomical MR imaging from brain hemodynamics [7] or mental operations to brain functions [8]. fMRI provides the ability to observe both the structures and also which structures participate in specific functions. fMRI provides high-resolution, noninvasive observation of neural activity. This ability to directly observe brain function opens good opportunities to advance our understanding of brain organization. This paper briefly introduces the fundamental principles of fMRI and some recent directions [2].

Integration of electroencephalography (EEG) and fMRI has been pursued in an effort to achieve greater spatiotemporal resolution of imaging dynamic brain activity [9]. Recently, simultaneous EEG-fMRI measurements have been used to investigate the relation between the two signals. Previous attempts at the analysis of simultaneous EEG-fMRI data reported significant correlations between regional BOLD activations and modulation of both event-related potential (ERP) and oscillatory EEG power, mostly in the alpha but also in other frequency bands [10]. Functional MRI has high spatial resolution but relatively poor temporal resolution (in seconds). EEG directly measures the brain's electrical activity, giving high temporal resolution (in milliseconds) but low spatial resolution. The two techniques are therefore complementary and may be used simultaneously to record brain activity. Recording an EEG signal inside an MRI system is technically challenging. The MRI system introduces artifacts into the EEG recording by inducing currents in the EEG. This can happen through several different mechanisms. An imaging sequence applies a series of short radiofrequency pulses which induce a signal in the EEG system. The pulses are short and relatively infrequent, so interference may be avoided by blanking the EEG system during the transmission. The EEG system also affects the MRI scan. Metal in the EEG leads and electrodes can introduce susceptibility artifacts into MR images. Care must be taken to limit currents induced in the EEG leads via the MRI system, which could heat the leads sufficiently to burn the subject [3].

Preliminary investigations of human brain mapping with these procedures have yielded insights into the functional organization of various sensory, motor, and language systems. In fact, BOLD effects are measured using rapid volumetric acquisition of images, with moderately good spatial and temporal resolution. Images are usually taken every several seconds, and the voxels in the resulting image typically represent cubes of tissue about several millimeters. Practically, the course of a BOLD response to a briefly presented stimulus lasts about 15 seconds for the robust positive response [3].

**Figure 1:**
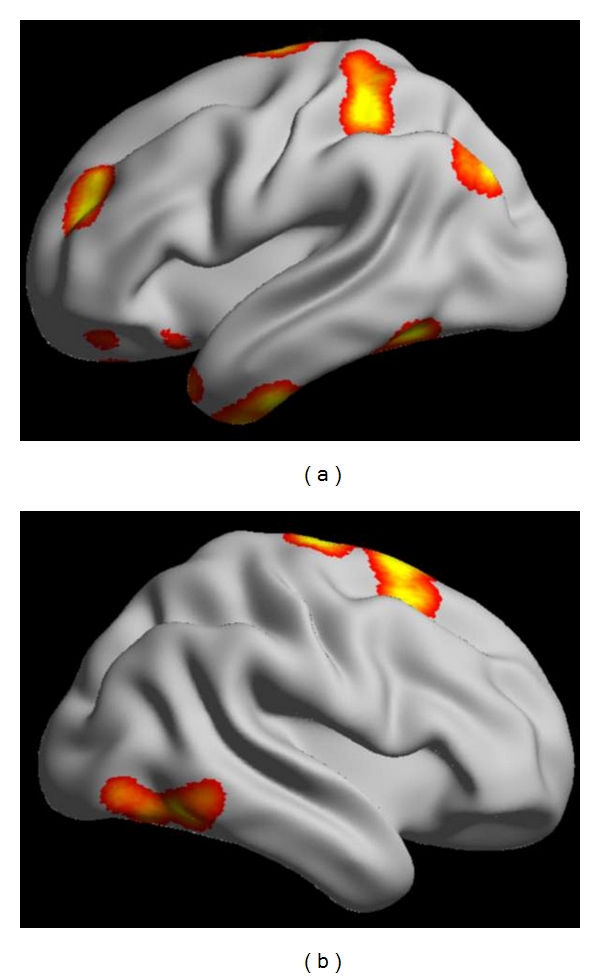
Reconstructed fMRI study for neural activity analysis.

A typical procedure of clinical applications includes these steps. (1) *Image Acquisition*. Images are acquired using a weighted gradient echo sequence. The system is equipped with echo planar options for rapid image acquisitions. Slice thickness is usually set at 3–5 mm. Simultaneous images are acquired on as many as tens of contiguous slices. (2) *Image Processing*. Some processing programs are developed as a stand-alone system outside of the scanner system. They provide the computational capability to reconstruct the large numbers of images and statistical analyses that identify the active areas. (3) *Task Procedure*. Patients are positioned in the scanner as for a conventional scan. During a functional imaging series, tens of images are obtained. To identify brain tissue involved in language, sensory, visual, auditory, hand movement, and other targeted functions, the patient acts accordingly during the activity epoch. The beginning and end of this activity period are cued by a visual or auditory signal. (4) *Data Analysis*. Statistical analyses are often used to identify areas of the brain activated by specific tasks and are based on a multistage comparison of stimulation and resting intensity levels as well as multiple replications.  Figure 1 shows a reconstructed fMRI study for neural activity analysis in our laboratory.

The scope of this paper is restricted to the most recent fMRI research for imaging neural activity in the human brain, mostly to introduce the research progress in 2010-2011. Although fMRI has attracted many researchers as early as since the 1980s, this paper concentrates on the most recent contributions. Furthermore, we include only what we believe to be representatives of important works and trends from recent years. The paper has four more sections.  Section 2 introduces the relevant aims and applications of neural activity analysis by fMRI.  Section 3 summarizes typical technologies in development.  Section 4 is a discussion and Section 5 is the conclusion.

## 2. Neural Activity from fMRI

### 2.1. Functional Connectivity

Functional connectivity measures based upon low-frequency BOLD fMRI signal fluctuations have become a widely used tool for investigating spontaneous brain activity. However, the precise relationship between neural activity, the hemodynamic response and fluctuations in the MRI signal is still unknown. Recent works had shown that correlated low-frequency fluctuations in the BOLD signal could be detected. Building on this preliminary work, Williams et al. demonstrate that functional connectivity observed in the rat depends strongly on the type of anesthesia used [11]. Lin et al. find that functional connectivity between cortical areas can be further revealed from the imaged source signals using phase synchrony measures [9]. Their approach is to image continuously oscillatory activities and their functional connectivity. Such ability promises to facilitate the investigation of the long-term neural behaviors and large-scale cortical interactions involved in spontaneous brain activity and cognition.

### 2.2. Biological Findings

**Figure 2:**
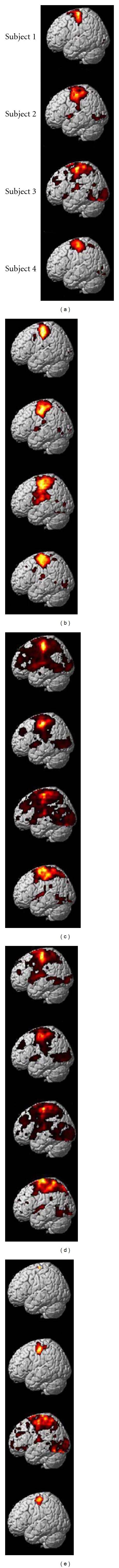
Comparative experiments using the design matrices constructed by (a) canonical HRF, (b) data-driven sparse GLM, (c) sICA using Infomax, (d) sICA using FastICA, and (e) PCA (Lee et al. 2011 [12] (2011 IEEE)).

Sparsity of the signal has been shown to be more promising in [12]. This coincides with biological findings such as sparse coding in the primary visual area (V1) simple cells, electrophysiological experiment results in the human medial temporal lobe, and so forth A data-driven fMRI analysis is derived solely based upon the sparsity of the signals [12]. Comparative experiments have been done using canonical HRF, data-driven sparse GLM, sICA using Infomax, sICA using FastICA, and PCA (Figure 2). Khadka et al. attempted to find neural correlates between the performed cognitive tasks and hemodynamic signals detected by a diffuse optical tomography system [1]. The initial observation showed activation of oxyhemoglobin concentration in Brodmann area 10 (BA10), which is consistent with some results seen by positron emission tomography (PET) and fMRI.

Many studies assume a simple relationship between neural and BOLD activity, in spite of the fact that it is important to elucidate how the “when” and “what” components of neural activity are correlated to the “where” of fMRI data. Murayama et al. conducted simultaneous recordings of neural and BOLD signal fluctuations in V1 cortex of anesthetized monkeys. They explored the neurovascular relationship during periods of spontaneous activity. The results showed a positive neurovascular coupling with a lag of 4-5 seconds and a larger contribution from local field potentials (LFPs) in the gamma range than from low-frequency LFPs or spiking activity. The method also detected a higher correlation around the recording site in the concurrent spatial map, even though the pattern covered most of the occipital part of V1 [6].

By integration of EEG source imaging and fMRI during continuous viewing of natural movies, it is found the most significant correlations in visual area V1. By calculating the impulse response function (IRF) between the BOLD signal and the estimated current density in area VI, it is found that the IRF is very similar to that observed using combined intracortical recordings and fMRI experiments in nonhuman primates. Taken together, these findings open an approach to noninvasive mapping of the brain. This is especially useful in combined EEG/fMRI experiments, where one can potentially study neural-hemodynamic relationships across the whole brain volume [13].

### 2.3. Vision and Hearing Research

In the community, fMRI has been applied for discovery of visual illusions, depth perceptions, hearing, or language-specific areas. Visual perceptual experiments have identified three types of neural pathways that represent color. It might be expected that there are neurons in the primary visual cortex that resemble the three perceptual pathways. Engel et al. used fMRI to examine responses in the human brain to a large number of colors. In visual cortical areas V1 and V2, the strongest response is to red-green stimuli, and much of this activity is from neurons receiving opposing inputs from L and M cones [14].

In [15], multivoxel pattern analysis is applied to investigate the specificity of brain activation patterns induced by acupuncture stimulations at a vision-related acupoint (GB37) and a nearby nonacupoint (NAP). Results showed that multiple areas could differentiate the neural response patterns induced by stimulation at the two sites with higher accuracy above the chance level [15]. Rauch et al. also studied effects of the local anesthetic Lidocaine on BOLD activity in V1 of nonhuman primates. Using independent component analysis (ICA), they describe and quantify the pharmacodynamics and spatial distribution of Lidocaine effects on visually evoked V1 BOLD signal in a dose-dependent manner [16].

In [17], fMRI was used to investigate differences in neural activity between subjects that can modulate their tinnitus by jaw protrusion and normal hearing controls. Lanting et al. measured responses to bilateral sound and responses to jaw protrusion. The auditory system responded to both sound and jaw protrusion [17]. In the experiments by [18], participants identified speech sounds masked by varying noise levels while blood oxygenation signals were recorded with fMRI. Accuracy and response time were used to characterize the behavior of sensory and decision components of the perceptual system.

### 2.4. Emotional Research

Emotion plays a significant role in goal-directed behavior, but people yet know very little about its neural basis. In several psychological models the cardinal dimensions that characterize the emotion space are considered to be valence and arousal. In [19], 3T fMRI was used to reveal brain areas that show valence- and arousal-dependent BOLD signal responses. Influential theories of human emotion argue that subjective feeling states involve representation of bodily responses elicited by emotional events. Individual differences in intensity of emotional experience reflect variation in sensitivity to bodily responses. Critchley et al. measured regional brain activity by fMRI during an interoceptive task wherein subjects judged the timing of their own heartbeats. They observed enhanced activity in insula, somatomotor, and cingulate cortices [20].

### 2.5. Neurosurgical Planning

Since neurosurgery relies on a precise delineation of the structural and functional aspects of brain, the role for fMRI in neurosurgical planning is very significant. The need for individualized maps of brain function is enhanced when the presence of a tumor alters the expected location of a function or when the location of the tumor is in an area with an uncertain function. fMRI does provide a source of precise functional and structural information for neurosurgery [2]. Many examples illustrated the potential advantage of functional and anatomical information for surgical treatment of brain tumors.

Recently, a cohort of neurosurgical patients are participants in a protocol at Columbia to evaluate the potential applications of fMRI for neurosurgical planning. These patients receive a standard battery of tasks targeted to localize language, sensory, motor, and visual areas both as candidates for surgery and as postsurgical patients. The objective of this investigation is to determine the potential role of functional mapping for neurosurgical procedures [2].

### 2.6. Pain Management

The experience of chronic and persistent pain is a debilitating condition for which the role of cortical processing is not well understood. The ability to use environmental stimuli to predict impending harm is critical for survival. Such predictions should be available as early as they are reliable. Chains of successively earlier predictors are studied in terms of higher-order relationships and have inspired computational theories such as temporal difference learning. However, there is yet no adequate neurobiological account of how this learning occurs. In [8], by fMRI study of higher-order aversive conditioning, a computational strategy is described that humans use to learn predictions about pain.

People have focused on the identification of cortical areas that are modified by the reduction of pain following pain therapy. Recent studies indicate that the cortical representation of sympathetically maintained pain involves specific and identifiable cortical activity, as well as does the relief of that pain achieved by a peripheral nerve block procedure. The preliminary studies suggest a wide range of other approaches using fMRI to investigate cortical representations of specific pain types and, therefore, potential specific therapy options [2].

### 2.7. Others

With the ability to image the entire 3D volume of human brain, fMRI is capable of isolating many simultaneous and coordinated brain events. The multilevel view of brain activity can include executive functions and high-level cognitive tasks simultaneously with the inputs such as vision and audition. Methods can also be developed to identify brain structures involved with visual perception, language generation, comprehension of sequential information as in a video, the execution of visually guided responses, and problem solving. These aspects of brain function have not previously been scrutinized with such precision in neuroscience [2]. Many more applications can be explored. For example, Kannurpatti et al. studied neural and vascular variability and the fMRI-BOLD response in normal aging [21].

## 3. Processing Technology

### 3.1. Formulations and Basic Processing

Formulization and basic fMRI processing technologies are always useful to bring upgraded hardware and software. For example, fMRI is very sensitive to artifacts created by head motion and magnetic field deformation. It is thus necessary to attenuate these artifacts in order to obtain correct activation patterns. A model-based method is introduced in [22] to remove motion artifacts in short-duration movements. The algorithm can account for head movement and field deformations due to movements within and outside of the field of view [22]. On the other hand, the power of fMRI in assessing neural activities is hampered by intersubject variations in basal physiologic parameters, which may not be related to neural activation but has a modulatory effect on fMRI signals. Therefore, normalization of fMRI signals is useful in reducing variations and improving sensitivity [23].

The common gradient-echo echo-planar imaging technique in fMRI is sometimes hampered by macroscopic field inhomogeneities. This can affect the degree of signal change that occurs in the images as a response to neural activation and the subsequent blood oxygenation changes, that is, the BOLD sensitivity. In [24], quantitative sensitivity maps are calculated directly from gradient-echo field maps.

### 3.2. General Linear Model

To test for spatial heterogeneity, a direct statistical measure is proposed in [25] for the existence of distributed spatial patterns applicable to fMRI datasets. They extend the univariate general linear model (GLM) [12], typically used in fMRI analysis, to a multivariate case. Contrasting maximum likelihood estimations of different restrictions on this multivariate model can be used to estimate the extent of spatial heterogeneity. The test statistic is assessed using simulated time courses derived from real fMRI data followed by analyzing data from a real fMRI experiment [25].

### 3.3. Nonlinear Model

The signals and images acquired through this imaging technique demonstrate the brain's response to prescheduled tasks. Several studies on BOLD signal responses demonstrate nonlinear behavior for a stimulus. Taalimi and Fatemizadeh propose a mathematical approach for modeling BOLD signal activity, which is able to model nonlinear behaviors of physiological systems [26]. A nonlinear auto regressive moving average model is used to describe the mathematical relationship between output signals and predesigned tasks. Parameters can be used to distinguish between rest and active states of a brain region.

### 3.4. Generative Model

Generative models, such as the most typical ICA methods, can be used for the observed multivariate data in a large database of samples. ICA can separate a multivariate signal into additive subcomponents supposing the mutual statistical independence of the non-Gaussian source signals [16]. It is a special case of blind source separation and has been broadly applied to fMRI due to its capacity to separate spatially or temporally independent components. However, the assumption of independence has been challenged by recent studies, and, therefore, ICA does not guarantee independence of simultaneously occurring distinct activity patterns [12]. Missimer et al. compared two data-driven methods of statistical image analysis, principal component analysis (PCA) and ICA, in identifying neural networks related to the transient occurrence of phosphenes experienced by a patient subsequent to a brain infarct [27].

ICA of fMRI time series reveals distinct coactivation patterns in the resting brain representing spatially coherent spontaneous fluctuations of the fMRI signal. Among these patterns, the default-mode network has been attributed to the ongoing mental activity of the brain during wakeful resting state [28].

A data-driven approach is proposed in [9], which starts with using ICA to decompose the spatiotemporal EEG data into a linear combination of scalp potential maps and time courses. The time course of each independent component is used to construct a regressor to fit the fMRI time series. The resultant fMRI map then feeds back as a spatial constraint to the estimation of the source distribution underlying the corresponding component map. The estimated source distributions multiplied by the corresponding component time courses are summed across all components, giving rise to the reconstructed spatio-temporal activity [9].

### 3.5. Spatial Pattern Analysis

Much current works in fMRI employ multivariate machine-learning approaches (e.g., support vector machines, SVMs) to detect distributed spatial patterns from the temporal fluctuations of the neural signal. The aim is not classification, but investigation of multivariate spatial patterns, which pattern classifiers detect only indirectly [25]. These analyses demonstrate the utility of the measure of heterogeneity as well as considerations in its application. Measuring spatial heterogeneity in fMRI may have potential uses for better characterising neurological conditions such as stroke and Alzheimer's disease [25].

By combination of simultaneous EEG-fMRI imaging information and using multivariate machine-learning-based regression, De Martino et al. find that it is possible to predict EEG power oscillations from simultaneously acquired fMRI data during an eyes-open/eyes-closed task using either the original channels or the underlying cortically distributed sources as the relevant EEG signal for the analysis of multimodal data [10].

The cause of the detected brain activity relies on the anatomy. Diffusion tensor MR imaging as a noninvasive modality providing in vivo anatomical information allows determining neural fiber connections which leads to brain mapping. The main drawback of reliable fiber mapping is the correct detection of the orientation of multiple fibers within a single imaging voxel. Duru and Ozkan propose a method based on linear data structures to define the fiber paths regarding their diffusivity [29]. 

### 3.6. Statistical Analysis

Statistical methods are a valuable tool for decoding information from neural imaging data [30]. The noisy signal and the limited number of training patterns that are typically recorded from fMRI pose a challenge for the application of statistical learning methods in data analysis. For a typical fMRI scan, the 3D volume of the head is imaged every one or two seconds, producing a few hundred to a few thousand complete images per scanning session. Because of practical limitations of the scanner, small motions on the part of the subject and the subject's pulse and respiration will affect the images. After reconstruction, the scanning session consists of a series of 3D images. The most common tasks to perform on these images are corrections for motion and physiological effects. Outlier removal and spatial and temporal filtering may be performed. A variety of methods are used to correlate the voxel time series with the task to produce maps of task-dependent activation [3]. Multivariate statistical analysis is applied to compare multivariate data and establish the quantitative changes and differences between groups under investigation on their characteristics. PCA displays the original variables in a space, thus reducing the dimensionality of the data and allowing the visualization of a large number of variables.

Kangjoo et al. propose a statistical analysis method for fMRI to overcome the drawbacks of conventional data-driven ICA. A compressed sensing-based data-driven sparse GLM is proposed that enables estimation of spatially adaptive design matrix as well as sparse signal components that represent synchronous, functionally organized and integrated neural hemodynamics. Furthermore, a minimum-description-length (MDL-) based model order selection rule is shown to be essential in selecting unknown sparsity level for sparse dictionary learning. Lee et al. propose a method that can adapt individual variation better than the conventional ICA methods do [12]. In fact, statistical parametric mapping is applied with a GLM expressed as

(1) $$y_{i}=Dx_{i}+\varepsilon_{i},\;i\in \left[1,N\right],$$

where **D** denotes the regressors and **x**_*i*_ denotes the corresponding response signal strength at the *i*th voxel. In the standard GLM model, the design matrix **D** is a predefined matrix. In [12], the data-driven sparse GLM assumes **D** as an unknown *global *dictionary, of which atom is assumed to indicate a principally dominant neural response in a small set of synchronous neural dynamics.

Danmei et al. propose using prior knowledge based on the behavioral performance of human observers to enhance the training of SVMs. They collect behavioral responses from human observers performing a categorization task during fMRI scanning and use the psychometric function generated based on the observers behavioral choices as a distance constraint for training an SVM. It is found that the behavior-constrained SVM outperforms SVM consistently [30], where the discrimination function is expressed as

(2) f(x)=wx+b.

Its parameters are optimized through minimizing a cost function. In nonlinear cases, the solution is reformulated as

(3) $$f\left(x\right)=\underset{i\in SV}{\sum }a_{i}y_{i}K\left(x_{i},x\right)+b,$$

where **K**(**x**_*i*_, **x**) is the kernel function.

The ultimate goal of fMRI data analysis is to detect correlations between brain activation and the task the subject performs during the scan. The BOLD signature of activation is relatively weak, however, so other sources of noise in the acquired data must be carefully controlled. A series of processing steps must be performed on the acquired images before the actual statistical search for task-related activation can begin [3].

### 3.7. Correlation Analysis

The precise relationship between neural signals and BOLD is an open problem. In general, changes in BOLD signal are well correlated with changes in blood flow. Numerous studies during the past decades have identified a coupling between blood flow and metabolic rate, that is, the blood supply is tightly regulated in space and time to provide the nutrients for brain metabolism [3].

Murayama et al. explored the neurovascular relationship during periods of spontaneous activity by using temporal kernel canonical correlation analysis, which is a multivariate method that can take into account any features in the signals that univariate analysis cannot. The method detects filters in voxel space and in frequency time space that maximize the neurovascular correlation without any assumption of a hemodynamic response function. The results are consistent with those of previous studies and represent the multivariate analysis of intracranial electrophysiology and high-resolution fMRI [6].

To analyze EEG data in natural setting, Whittingstall et al. developed an ICA-based method to reject EEG artifacts due to blinks, subject movement, and so forth. They calculate the EEG source strength of the artifact-free data at each time point of the movie within the entire brain volume using low-resolution electromagnetic tomography. This provided for every voxel in the brain an estimate of the current density at every time point. A correlation is carried out between the time series of visual contrast changes with that of EEG voxels [13].

While current data indicate that local field potentials, an index of integrated electrical activity, form a marginally better correlation with blood flow than the spiking action potentials that are most directly associated with neural communication [15], no simple measure of electrical activity yet can provide an adequate correlation with metabolism and the blood supply across a wide dynamic range. Presumably, this reflects the complex nature of metabolic processes, which form a superset with regards to electrical activity. An initial small, negative dip before the main positive BOLD signal is more highly localized and also correlates with measured local decreases in tissue oxygen concentration. One problem with this technique is that the early negative BOLD signal is small and can only be seen using larger scanners with magnetic fields of at least 3 Tesla. Further, the signal is much smaller than the normal BOLD signal, making extraction of the signal from noise very difficult. Also, this initial dip occurs within 1-2 seconds of stimulus initiation, which may not be captured when signals are recorded at long repetition [3]. An event-related fMRI study was designed in [31] to dissociate the neural correlates of two putative key functions, volitional saccade generation and inhibition of reflexive saccades, and to investigate their interaction.

### 3.8. Multimodal Combination

Multimodal imaging techniques rely on the assumption of a common neuronal source for different recorded signals. In order to maximally exploit the combination of these techniques, we need to understand the coupling between EEG and fMRI BOLD signals. Beyond the correlation of the two measured brain signals, the ability of predicting the signal in one modality using information from the other modality is studied in [10]. Lin et al. report a data-driven approach to image spatio-temporal features of neural oscillatory activity and event-related activity from continuously recorded EEG and fMRI signals [9]. Lanting et al. studied multimodal integration of somatosensory jaw protrusion and sound [17]. Having simultaneously recorded EEG and fMRI data, the final hurdle is to coregister the two datasets, as each is reconstructed using a different algorithm, subject to different distortions [3].

By combining the neural and hemodynamic recordings in multiple modalities, we can get better insight into how and where the brain processes complex stimuli, which is especially useful for dealing with different neural diseases. However, due to different spatial and temporal resolutions, the integration of EEG and fMRI recordings is not straightforward. One fundamental obstacle is that paradigms used for EEG experiments are usually event related, while fMRI is not limited in this regard. Furthermore, integration of the EEG signal directly with the BOLD signal is useful, by estimating the underlying IRF that relates the BOLD signal to the underlying current density [13].

## 4. Discussion

The main advantages of fMRI to image brain activity related to a specific action or sensory process include. (1) The signal does not require injections of radioactive isotopes. (2) The total scan time required can be very short, for example, in 1-2 minutes. (3) The in-plane resolution of the functional image is generally about 1 × 1 mm^2^. To put these advantages in perspective, functional images obtained by the earlier PET method require injections of radioactive isotopes, multiple acquisitions, and long imaging times. Further, the resolution of PET images is much larger than that in usual fMRI images. Additionally, PET usually requires that multiple individual images are combined in order to obtain a reliable signal. Consequently, information on a single patient is compromised and limited to a finite number of imaging sessions, and thus it is not optimally suitable to assist in a neurosurgical or treatment plan for specific individuals [2].

Many difficulties still exist in the fMRI technology. The images produced must be interpreted carefully, since correlation does not imply causality, and brain processes are complex and often nonlocalized. Statistical methods must be carefully used because they can produce false positives. The BOLD signal is an indirect measure of neural activity, which is susceptible to be influenced by nonneural changes in the body. It is difficult to interpret positive and negative BOLD responses. BOLD signals are most associated with the input to a given area rather than with the output. It is possible that a BOLD signal exists in an area even without a concrete activity there.

The BOLD response reaches to a peak in about 5 seconds after neuronal firing begins and fMRI has poor temporal resolution. It is, therefore, difficult to tell BOLD responses with different events occurring in a short period, although good experimental design can reduce this problem. Some researchers attempt to combine fMRI signals that have relatively high spatial resolution with signals recorded with other techniques, for example, EEG or magnetoencephalography (MEG), which have higher temporal resolution but worse spatial resolution. fMRI has often been used to show activation localized to specific regions, thus minimizing the distributed nature of processing in neural networks. The BOLD response can be affected by a variety of factors, for example, drugs, substances, age, brain pathology, local differences in neurovascular coupling, attention, amount of carbon dioxide in the blood, and so forth. For these reasons, functional imaging provides insights into neural processing that are complementary to insights of other studies in neurophysiology [3].

The aim of this paper is to introduce the most recent work of fMRI and potential future applications, for example, neurosurgical planning and risk assessment, strategies for the treatment of chronic pain, seizure localization, and understanding of the physiology [2]. Although fMRI has been developed in many years as a relatively mature approach to estimation and diagnosis, problems still exist in its analysis in biomedical engineering. Researchers are exerting efforts in improving all simple and complex aspects.

## 5. Conclusion

Functional magnetic resonance imaging has been widely-used for detection of the brain's neural activity. This paper summarizes the annual progress for biomedical applications. Typical contributions are addressed for biological findings, functional connectivity, vision and hearing research, emotional research, neurosurgical planning, pain management, and so forth. Representative contributions are listed to show a general overview of new results in 2010-2011. Processing technology for solving fMRI problems is summarized. Particularly, introduced models and strategies include general linear model, nonlinear model, generative model, spatial pattern analysis, statistical analysis, correlation analysis, and multimodal combination.
